# Supplementary material for: A Soil-Isolated Streptomyces spororaveus Species Produces a High-Molecular-Weight Antibiotic AF1 against Fungi and Gram-Positive Bacteria
Source: Antibiotics (Basel). 2022 May 18;11(5):679. doi: 10.3390/antibiotics11050679 (PMC9138071; doi:10.3390/antibiotics11050679)
Supplement: Supplementary file 1 [file antibiotics-11-00679-s001.zip › antibiotics-1659614-supplementary.pdf]

antibiotics

MDPI

Supplementary Materials:

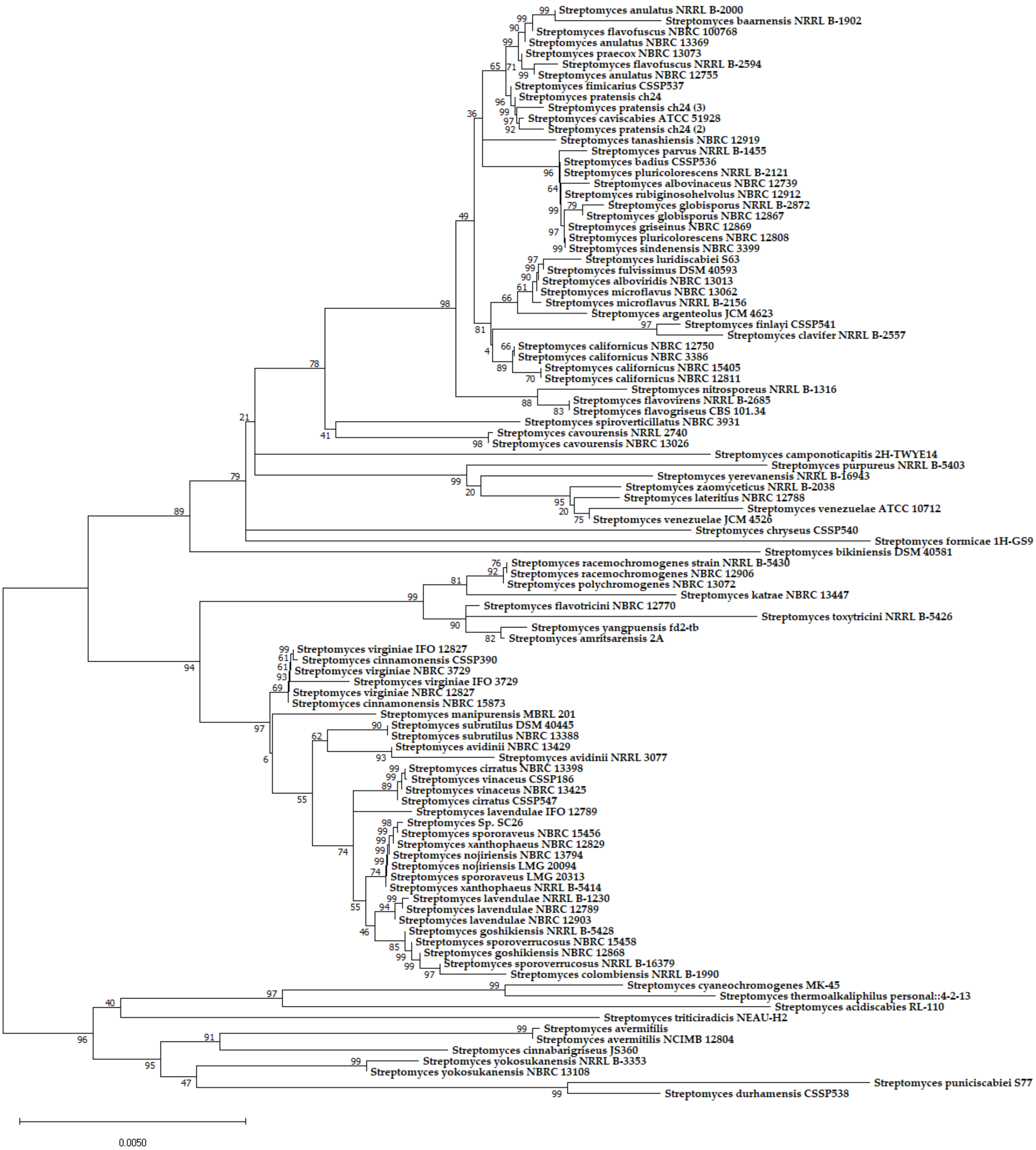

Figure S1. Phylogenetic tree of 16S ribosomal RNA of SC26. Neighbor-joining method was conducted based on 16S rRNA gene sequences from 100 *Streptomyces*. The bootstrap consensus tree which inferred from 1000 replicates is drawn to scale, with branch lengths, indicating the number of substitutions per site between species using MEGA 11.

Table S1. Genome Features of SC263.

Feature		Characteristics
Number of Contigs		3
Total GC Content		71.92%
Length (base pair, bp)		8,217,694
	Ctg1	7,201,269
	Ctg2	996,243
	Ctg3	20,182
Number of Genes		7,370
Number of RNAs		91
	rRNA	21
	tRNA	73
	ncRNA	3
Number of Subsystems		319
Number of Pseudogenes		197
Number of BGCs		28 (see Table S2)

Table S2. Biosynthetic Gene Clusters of *S. spororaveus* SC263 and NBRC 15456

BGC Type	Most similar known cluster	Notes	*S. spororaveus* SC263		*S. spororaveus* NBRC 15456	
			Region	Similarity	Region	Similarity
NRPS-like	lipstatin	NRP	1-1	42%	1	35%
T2PKS	spore pigment	Polyketide	1-2	66%	2	66%
NRPS, NRPS-like	JBIR-126	NRP	1-3	92%	3	96%
NRPS, T1PKS	coelichelin	NRP	1-4	72%	4	72%
butyrolactone	neocarzinostatin	Polyketide: type I	1-5	4%	5	4%
NRPS, T1PKS	versipelostatin	Polyketide	1-6	14%	6	8%
siderophore	desferrioxamin B	Other	1-7	100%	7	100%
phenazine	lomofungin	Other	1-8	34%	8	34%
LAP			1-9		9	
CDPS	BD-12	NRP	-	-	10	17%
siderophore	ficellomycin	NRP	1-10	5%	11	5%
T1PKS	ECO-02301	Polyketide	1-11	82%	12	82%
NRPS-like	meoabyssomicin / abyssomicin	Polyketide	1-12	12%	-	-
NRPS-like, T2PKS	polyketomycin	Polyketide: type I +Type II	1-13	39%	13	41%
RiPP-like			1-14		14	
terpene	toxoflavin / fervenulin	Other	1-15	14%	15	14%
lanthipeptide-class-iv			1-16		16	
NRPS	nogalamycin	Polyketide	1-17	30%	17	30%
terpene	hopene	Terpene	1-18	61%	18	61%
ectoine	ectoine	Other	1-19	100%	-	-
lanthipeptide-class-I			1-20		-	-
T1PKS, hglE-KS	saframycin A / saframycin B	NRP	1-21	4%	19	4%
lanthipeptide-class-iii, terpene	SapB	RiPP:Lanthipeptide	2-1	100%	20	100%
terpene	2-methylisoborneol	Terpene	2-2	100%	21	100%
terpene	monensin	Polyketide	2-3	5%	22	5%
melanin	melanin	Other	2-4	28%	23	28%
siderophore			2-5		24	
T3PKS	alkylresorcinol	Polyketide	2-6	100%	25	100%
CDPS, NAPAA			2-7		26	
